# CON: Procalcitonin does not have clinical utility in children with community-acquired pneumonia

**DOI:** 10.1093/jacamr/dlab152

**Published:** 2021-10-22

**Authors:** Ritu Banerjee

**Affiliations:** Vanderbilt University Medical Center, Nashville, TN, USA

## Abstract

Most clinical studies supporting procalcitonin (PCT)-guided management of lower respiratory tract infections have been performed in adults. There is a paucity of studies evaluating the clinical impact of PCT use in children and limited data informing age-appropriate PCT cut-offs; diagnostic accuracy in immunocompromised children; patient subgroups most likely to benefit from PCT testing; whether PCT adds value beyond available rapid molecular viral diagnostics; and optimal implementation strategies for PCT-guided treatment. At the present time there is little evidence to support routine use of PCT to aid management of paediatric pneumonia.

Procalcitonin (PCT) is a biomarker that shows promise in identifying bacterial infection and is increasingly used in patients of all ages. Several PCT assays are approved by the US FDA for prediction of mortality and to guide antimicrobial management in sepsis and lower respiratory tract infection (LRTI). However, the vast majority of clinical studies supporting PCT-guided management have been done in adults. Few rigorous, interventional studies have evaluated the impact of PCT use in children, in whom its clinical utility is unclear. At the present time there is little evidence to support routine use of PCT to aid management of paediatric pneumonia.

Despite extensive evaluation in adults, it is still not clear whether use of PCT provides benefit for management of adult LRTI. Schuetz *et al*.^1^ summarized 26 randomized controlled trials (RCTs) evaluating PCT use in a variety of LRTIs in adults. There were 3336 subjects in the PCT arm and 3372 subjects in the control arm. The primary outcome was initiation of antibiotics, which was significantly lower in the PCT versus control arm (70% versus 86%, *P *<* *0.001). However, more recently, the ProACT trial enrolled 1656 adults with LRTIs from 14 US emergency departments and randomized them to management using a PCT testing and treatment algorithm versus usual care. The primary outcome was antibiotic days by 30 days after enrolment.^2^ The authors found no significant differences between the groups in terms of antibiotic exposure, percentage of subjects receiving antibiotics in the emergency department and hospital length of stay. Notably, algorithm adherence varied and was as low as 39% for patients with community-acquired pneumonia (CAP), suggesting this is an important process outcome to measure in future studies. Among numerous RCTs evaluating the impact of PCT testing on antibiotic use for adults with LRTI, about half demonstrated reductions in antibiotic use with PCT while the remainder did not.^2–8^ Notably, no study published after 2016 demonstrated that PCT use was associated with less antibiotic use compared with usual care, perhaps because of better antibiotic stewardship or access to infectious disease diagnostics in the usual care group in recent years. For example, a recent RCT performed in several emergency departments in France found no difference in antibiotic exposure or outcomes whether adherence to clinical guidelines or a PCT-guided algorithm was used to manage adults with pneumonia.^9^ Reasons for discrepancies between these adult studies include differences in study populations, study designs and outcomes; timing and frequency of PCT collection; use of different PCT assays and cut-off values; and differences in compliance with PCT-based treatment algorithms. Given these variations, it is difficult to draw conclusions about PCT utility in adults or extrapolate from these studies to inform use of PCT in children.

In contrast to the numerous published adult LRTI PCT trials, there are only two paediatric RCTs evaluating PCT use in paediatric pneumonia. The ProPAED trial enrolled 337 subjects <18 years old with LRTIs in two emergency departments in Switzerland and randomized them to receive either PCT-guided antibiotic management or usual care.^10^ The authors found no difference in the antibiotic initiation rate between the groups, but found that antibiotic duration of therapy was shorter in the PCT group versus control group (4.5 versus 6.3 days, *P *=* *0.039). In another RCT, Esposito *et al*.^11^ randomized hospitalized children aged between 1 month and 14 years with uncomplicated CAP (defined as presence of respiratory symptoms and abnormality on chest radiograph) to receive either PCT-based treatment or usual care. One hundred and fifty-five subjects were enrolled in each group. The authors found that compared with the control group, the PCT group had significantly fewer antibiotic starts (100% versus 85%, *P *<* *0.05), and fewer antibiotic days (5.37 versus 10.96, *P *<* *0.05). Both the above trials enrolled subjects before 2010, prior to widespread use of molecular viral diagnostics; had a small sample size; did not include many subjects with severe CAP; and did not report compliance with the PCT algorithm, so the applicability of these studies to current practice is unclear.

Several observational studies highlight the limitations of PCT as a diagnostic aid for management of paediatric pneumonia. Twenty years ago a study of 72 children hospitalized with CAP found greater positive and negative predictive values of PCT than other biomarkers, including C-reactive protein and WBC count, for differentiating bacterial and viral causes of pneumonia.^12^ However, since then, data have been conflicting. In a retrospective evaluation of PCT concentrations among 532 hospitalized children with CAP who were enrolled in the Etiology of Pneumonia in the Community (EPIC) study, a PCT cut-off of 0.25 ng/mL demonstrated a sensitivity of 85% and a specificity of only 45%.^13^ Furthermore, in that study, 65% of children with a PCT concentration of ≥0.5 ng/mL had a viral or atypical pathogen (but no bacteria) detected as the cause of pneumonia. This cut-off point is above the 0.25 ng/mL PCT concentration used in the paediatric RCTs described above and raises concerns about what the appropriate cut-off point for PCT should be in children. A PCT cut-off point that is too low may lead to unnecessary antibiotic use in children. This finding also highlights the lack of specificity of PCT to accurately distinguish between bacterial and non-bacterial causes of pneumonia, particularly in children who often have viral LRTI.^14^^,^^15^ Florin *et al.*^16^ evaluated PCT diagnostic accuracy in a prospective cohort study of 477 children aged 3 months to 18 years who presented to an emergency department and were diagnosed with CAP. The authors found that PCT was not useful in discriminating non-severe from severe CAP. While the study did observe that CRP and PCT were higher in children with serious outcomes, including empyema and chest-drainage procedures, it is likely that clinical assessment alone would have identified children with these complications of CAP. A recent meta-analysis of 21 observational studies including 2864 paediatric patients evaluated PCT accuracy for identifying bacterial pneumonia (defined as presumptive or proven identification of pathogens causing typical and/or atypical bacterial infection using culture, PCR, antibody or antigen assays) and concluded that PCT had only moderate diagnostic accuracy with a combined sensitivity of 64% and specificity of 72%. Notably, studies in this meta-analysis utilized a wide range of PCT cut-offs, from 0.12 to 35.8 ng/mL,^14^ again raising concern about non-standardized PCT cut-offs.

Additional paediatric-specific evaluations of PCT in LRTI are clearly needed to address numerous questions about PCT, including determining appropriate age-based PCT cut-offs, diagnostic accuracy in immunocompromised children, and patient subgroups most likely to benefit from PCT testing. In some studies of paediatric sepsis and LRTI, low PCT values may reduce antibiotic use among patients with low acuity illness and low likelihood of having bacterial infection; thus, PCT may be most promising for ruling out rather than ruling in bacterial disease, but further studies are needed to confirm this.^16–19^ The optimal timing and frequency of PCT collection is also not clear. Use of serial PCT levels to guide antibiotic cessation has been done in clinical trials but is difficult to perform in real-life clinical practice in children. In the ProPICU trial, only 38% of patients had all four serial PCT levels drawn per protocol.^18^ The role of PCT used in conjunction with widely available rapid molecular viral diagnostics must be assessed. PCT may add incremental, if any benefit, beyond viral detection, especially with increasing provider awareness of the importance of antibiotic stewardship and reducing unnecessary antibiotic use for patients with viral infections. Cost-effectiveness analyses should be performed because if PCT does not add benefit, then its use increases cost without improving quality of care. Utilizing implementation science methods to ensure optimal PCT test uptake, interpretation and compliance with treatment algorithms is also critical. Pairing PCT testing with oversight by an antibiotic stewardship programme shows promise for increasing compliance with treatment algorithms, but this requires multidisciplinary collaboration and personnel which are not always feasible in all settings.^18^

In conclusion, the clinical utility of PCT to guide diagnosis and antibiotic management of paediatric pneumonia is unproven due to a paucity of paediatric studies. Even in adult populations, where numerous evaluations of PCT have been conducted, results are conflicting regarding whether use of PCT provides clinical benefit. There are several questions around PCT implementation and impact in paediatrics that are promising areas for future research. The existing body of evidence is not convincing that PCT adds anything beyond what other, cheaper biomarkers (like C-reactive protein) or clinical assessment can provide for management of paediatric pneumonia.

## Transparency declarations

None to declare.
